# Translation of Animal Study to Human: In Silico Based Development of Implantable Pulmonary Artery Pressure Sensor

**DOI:** 10.1002/cnm.70050

**Published:** 2025-06-02

**Authors:** Leonid Goubergrits, Pavlo Yevtushenko, Adriano Schlief, Jan Romberg, Titus Kuehne, Andreas Arndt, Jan Bruening

**Affiliations:** ^1^ Institute of Computer‐Assisted Cardiovascular Medicine Deutsches Herzzentrum der Charité Berlin Germany; ^2^ Biotronik Berlin Germany; ^3^ Technische Universität Dresden Dresden Germany

**Keywords:** animal trial, computational fluid dynamics, pressure sensor, pulmonary artery, thrombus formation

## Abstract

Implantable pulmonary artery pressure sensors (PAPS) might impose a flow‐induced risk of thrombus formation in the pulmonary artery (PA). To assess this risk, an in silico study‐enhanced animal study with 20 sensors implanted in 10 pigs had previously been conducted. In the in silico study, PAPS were virtually implanted mimicking real implantations, based upon data acquired by CT. This animal in silico study investigated changes in hemodynamics caused by PAPS using image‐based computational fluid dynamics (CFD). However, porcine and human PA differ significantly in geometry and hemodynamics. To investigate the transferability of animal in silico study findings toward human conditions, we propose a parallel in silico human study. Based on a similarity analysis (L1 norm for 8 geometric features) human PA geometries with the least difference to 10 porcine PA were selected. PAPS were virtually implanted in human PA as close as possible, mimicking the implantation configuration of the animal study. Finally, a numerical flow analysis of the hemodynamic changes due to PAPS implantation was done. Comparing human and porcine PA, we found significantly larger left and right PA diameters in humans, whereas no differences were found for main PA diameters and bifurcation angle. Comparing hemodynamic boundary conditions, we found a significantly smaller heart rate and a significantly higher peak systolic main PA flow rate in humans, whereas no significant differences for cardiac output were found. The human in silico PAPS study found no relevant changes in hemodynamics increasing the risk of thrombus formation after sensor implantation. This is also valid for PAPS that were non‐optimally implanted. Thus, despite differences between species, findings of the in silico animal study were confirmed by the human in silico study.

## Graphical abstract

The CFD study investigated the impact of the pressure sensor implanted in the porcine or human pulmonary artery (PA) on hemodynamics, aiming to prove the transferability of animal trial results to human conditions. We found that despite differences between human and porcine PA regarding morphometry and flow conditions, the results of the animal study are also valid for human conditions.

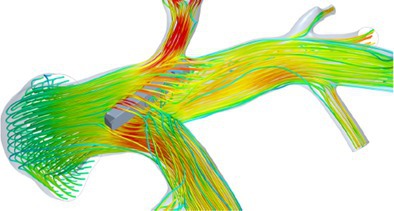

## 1 Introduction

The development of novel as well as further development of existing medical devices is a longstanding process including multiple steps, of which many might have to be repeated. According to the U.S. Food and Drug Administration (FDA), the development process usually starts with an ideation and prototyping phase [1]. After this initial phase, preclinical in vitro and animal testing is usually conducted to answer basic questions regarding device efficacy and safety.

Although animal models have always been used in basic research and medical device development to ensure safety and efficacy of medical devices before their use in humans, animal testing is subject to relevant criticism regarding, among others, the underlying ethical implications as well as the extent to which they are suited in replicating human conditions (transferability). Regarding the ethical implications, the 3Rs principle (replacement, reduction, and Refinement) has been elaborated in 1959 [2] laying the foundation for increasing focus on animal welfare and well‐being, which, among others, resulted in strict regulatory frameworks regarding the use of animal models in all stages of the medical device development cycle. The transferability of animal study results to human application has been repeatedly doubted due to marked differences in anatomy and pathophysiology between animal models and humans [3, 4, 5]. Even in surgical research, where interspecies differences are less relevant, results obtained via animal models are often not confirmed in human application [5].

A promising approach in the context of medical device development, including the 3R concept and the challenge of the transferability of animal study results, is the use of in silico models employing computational simulation approaches, rather than benchtop testing (in vitro) or animal studies (in vivo). In silico approaches, which have been increasingly used during the last two decades, promise to model different aspects of healthy or pathologic systems even in a patient‐specific manner as well as to support evaluation and approval of medical devices [6, 7, 8]. A potential application of in silico models is the calculation of hemodynamic parameters associated with device thrombosis. Implanted cardiovascular devices are associated with an elevated risk of thrombosis formation due to various aspects, such as mechanical, chemical, and biological interaction with foreign materials and disturbed and altered flow conditions [9].

Accordingly, the Horizon 2020 Research and Innovation Action funded SIMCor project (www.simcor‐h2020.eu) elaborated and evaluated the capabilities of in silico models to assess the safety and efficacy of cardiovascular implantable devices. One use case investigated in the SIMCor project is a novel, recently introduced class of implantable medical devices, the pulmonary artery pressure sensor (PAPS) [10]. PAPS allows measurement and monitoring of the pulmonary artery pressure (PAP). They have been introduced to improve the pharmacological management of heart failure (HF) patients and reduce hospital readmissions resulting from acute decompensation. HF is a leading cause of death and hospitalization with a high prevalence of 1%–2% [11], which is also associated with different disease causes and co‐morbidities [12]. Due to the poor prognosis of HF patients after hospitalization as well as the resulting significant costs and burden on healthcare systems, the development of novel methods for a reduction of hospital readmission is an ongoing process [13]. It was demonstrated that telemonitoring allows improving management of HF patients [14]. Furthermore, biomarkers such as pulmonary artery pressure are known to allow early prediction of deterioration of HF, such as acute decompensation. However, the measurement of PAP can usually only be conducted in a clinical setting via invasive catheterization. PAPS have been introduced as an alternative solution facilitating PAP measurement at home or in outpatient settings [10]. Thus, measurement of elevated PAP using PAPS is used for the prediction of adverse events, and a timely intervention via pharmaceutical treatment was shown to reduce the rate of hospitalizations [15]. Currently, the CardioMEMS HF‐System (Abbott) is the only device available in clinical routine [16, 17], whereas the Cordella HF system (Endotronix Inc) [18, 19] recently received FDA pre‐market approval for the U.S. market based on the PROACTIVE HF clinical trial [20]. Both systems differ in the recommended implantation site, slightly differ in the size of the sensor body (e.g., 15 mm length of the CardioMEMS vs. 19.3 mm length of the Cordella) and markedly differ in the recommended vessel diameter range of the implantation site (7–11 mm for the CardioMEMS vs. 12–26 mm for the Cordella). Clinical trials of the CardioMEMS system provide promising results regarding PAPS supported management of HF patients [21, 22].

A PAPS designed to be implanted in the left or right PA with target vessel diameters of about 10 mm is currently under development [23]. This includes animal experiments aiming to investigate various aspects including the feasibility and safety of the implantation procedure, fixation, durability, function, and the risk of device‐related complications, such as device thrombosis and lung embolism, device migration, as well as perforation of the arterial wall by the fixation elements. Since animal experiments are limited with respect to the information and parameters that can be measured in vivo, we proposed to use an in silico model to provide additional information on hemodynamics, thus refining the animal study.

An in silico study enhancing chronic animal study, in which the novel PAPS device was investigated, has been recently published [23]. In this study, hemodynamic parameters associated with device thrombosis, which cannot be measured in vivo, have been calculated. Intra‐arterial hemodynamics before and after device implantation in animals were simulated using computational fluid dynamics (CFD) based on subject‐specific information acquired by computed tomography (CT). The study compared hemodynamic parameters in the PA before and after virtual PAPS implantation and analyzed the effect of non‐optimal device positioning within the PA (skewed devices lying across the cross‐section of the vessel or devices being positioned in a side branch of the left or right PA). Non‐optimal device positioning results in disturbed, non‐physiologic flow conditions with stagnations or recirculation regions possibly increasing the risk of thrombus formation [24]. Results have been compared against clinical outcomes observed in animal experiments, providing additional evidence for the safety of the device with respect to the clinical endpoint of thrombosis. Translation of this study's results towards an application in humans is challenging since anatomy and consequently hemodynamic conditions differ among different species [25].

Therefore, the study presented here aims at assessing hemodynamic parameters associated with the risk of device thrombosis after PAPS implantation in the human PA. The study is aligned to the previously conducted animal in silico study briefly described above [23]. The study aims to assess whether the limited hemodynamic effects that have been observed in an animal application can be replicated in humans despite the differences in anatomy and hemodynamics.

## 2 Materials and Methods

This study was designed to complement an in silico study, which was used to enhance a chronic animal study conducted at the animal research facility of the Charité—Universitätsmedizin Berlin. This chronic animal study was approved by the relevant competent authority, the Regional Office for Health and Social Affairs Berlin (registration number G 0091/21), and was described in detail here [23]. Briefly, 20 PAPS were implanted in 10 pigs with an approximate weight of 60 kg on the day of implantation. Two sensors were implanted into each animal, one each into the left and right PA.

CT acquisition was done for each animal before implantation, immediately after, as well as at 30 and approximately 60 days after implantation. Details regarding implantation procedure, including sedation and anesthesia, CT imaging (device and contrast agent administration), and finally euthanasia followed by device explantation were published earlier [23].

To investigate porcine PA hemodynamics in 10 pigs with and without PAPS, PA surfaces, including main (MPA), left (LPA), and right (RPA) pulmonary artery, were reconstructed from CT data acquired before PAPS implantation using a mainly manual procedure with semi‐automatic steps using ZIBAmira (v. 2015.28, Zuse Institute Berlin, Germany). LPA and RPA were reconstructed together with their immediate side branches. More details on the segmentation and reconstruction of surfaces can be found here [23, 25]. Due to the presence of metallic artifacts in the post‐operative image data, PA geometries after device implantation could not be reconstructed from their respective image data. Instead, post‐intervention geometries were constructed by virtually implanting 3D CAD geometries of the PAPS into pre‐operative PA surfaces using post‐operative CT data, acquired 30 days after implantation, for PAPS positioning. The procedure of virtual PAPS implantation has been described in detail earlier [23]. Briefly, each PAPS geometry was manually translated and rotated to match the probe position found in the CT data (Figure 2B). For the human matches, probes were positioned at the same distance along the LPA/RPA centerline as their porcine counterpart and rotated to mimic a respective optimal/non‐optimal implantation position as defined further below. After positioning, a connected surface geometry enclosing the entire blood pool was generated by removing the volume enclosed by the PAPS geometries from the PA lumen via Boolean subtraction.

**FIGURE 1:**
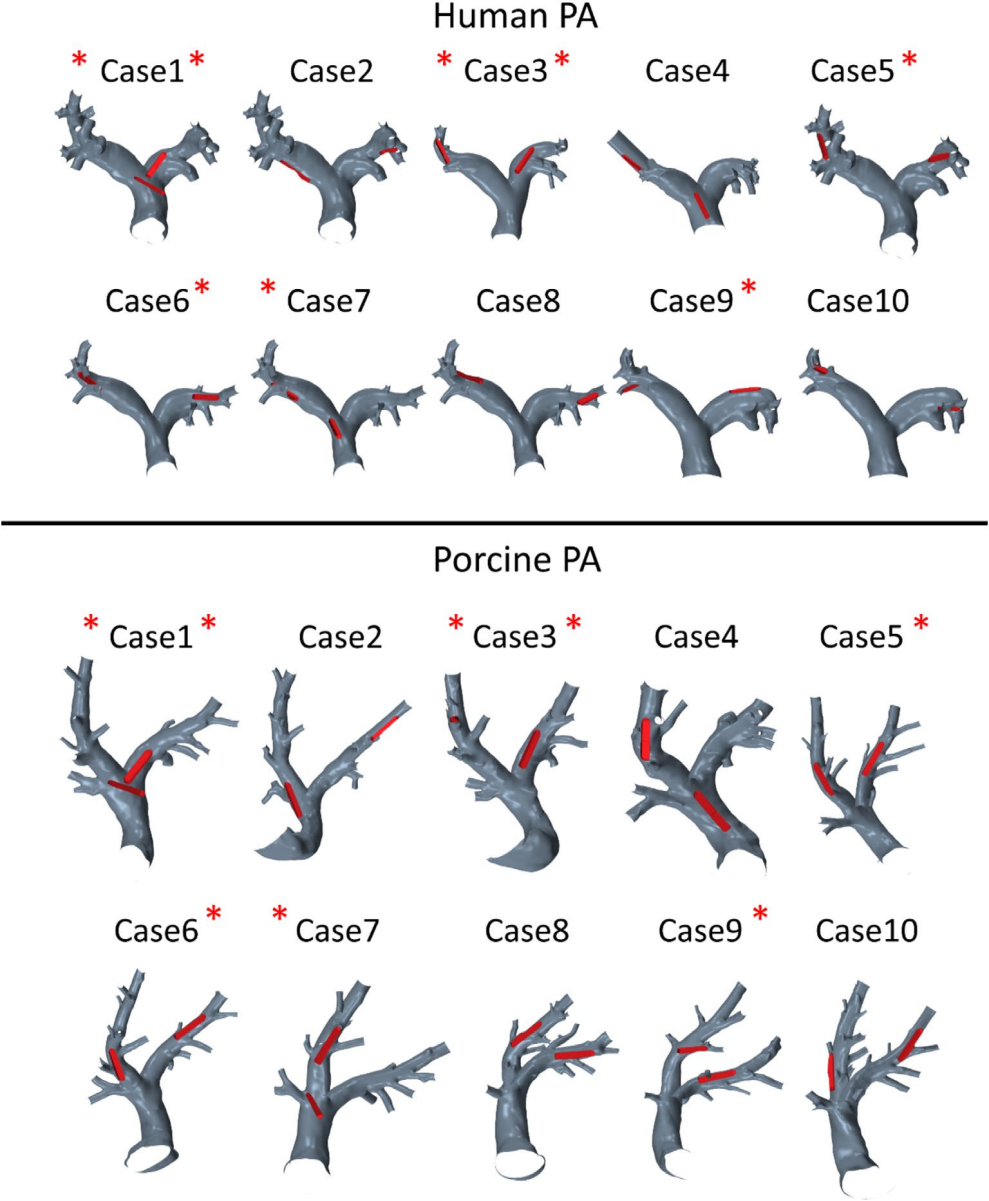
Geometries of 10 porcine PA and 10 human PA with virtually implanted PAPS. Cases with non‐optimally implanted sensors in the left and/or right pulmonary arteries are marked with a red asterisk (two asterisks if both sensors are non‐optimal). Note that case numbers reflect corresponding paired PA geometries (i.e., human case 1 is the match to porcine case 1 and so far).

To compare PA hemodynamics in porcine and human PA, suitable geometries were selected from an available retrospective cohort of 49 human PA, which was described and published earlier [25]. To each of the 10 porcine PA geometries a respective paired human PA geometry was selected from this cohort based on a similarity of geometric parameters. The parameters used for the similarity‐based selection were the lengths and diameters of all three major PA segments (MPA, LPA, and RPA), the LPA‐RPA bifurcation angle, and the number of side branches. Matching was performed based on an L1‐norm of the aforementioned geometric parameters. For each human geometry available, a similarity distance $S_{i}$ to a target porcine geometry is defined as:

$$S_{i}=\sum_{j=1}^{N}\left({P_{j}}_{i}-{P_{j}}_{T}\right)$$

where ${P_{j}}_{i}$ is the *j*‐th geometrical parameter (*N* = 8) of the *i*‐th human PA geometry and ${P_{j}}_{T}$ is the *j*‐the geometrical parameter of the *T*‐th target porcine surface. For each of the 10 porcine target geometries, the human geometry with the lowest similarity distance was chosen as the respective match and assigned the same case number (i.e., human case 1 is the match to porcine case 1 and so far). Furthermore, a maximal difference of 25% for each single geometrical parameter was allowed. However, since the anatomical variation of the 10 porcine PA cases was relatively low (see Table 1), some human PA cases have been the best anatomical match for several porcine PA (see Figure 1): porcine PA cases 2 and 5 are represented by the same human PA case, porcine PA cases 6, 7, and 8 are represented by a one human PA, as well as porcine PA cases 9 and 10 are represented by one human PA geometry. Thus, this study was conducted using only five different human PA shapes. The averaged values and standard deviations of these geometric parameters for both in silico cohorts are provided in Table 1.

**TABLE 1:** Comparison of geometric parameters between pig and human pulmonary arteries used in in silico studies: Length *L* and diameters *D* of three major PA branches, total number of LPA and RPA side branches N_SB and LPA–RPA bifurcation angle *α*.

Cohort	*L*_MPA_, mm	*D*_MPA_, mm	*L*_RPA_, mm	*D*_RPA_, mm	*L*_LPA_, mm	*D*_LPA_, mm	N_SB	α (°)
Pig	67 ± 9.9	23.8 ± 2.2	114 ± 14.3	14.9 ± 2.0	102 ± 12.2	13.6 ± 1.7	12.7 ± 2.3	80 ± 7
Human	40 ± 6.5	26.8 ± 3.9	90 ± 17.8	20.0 ± 1.2	97 ± 19.2	18.2 ± 1.3	11.4 ± 1.2	89 ± 8

**FIGURE 2:**
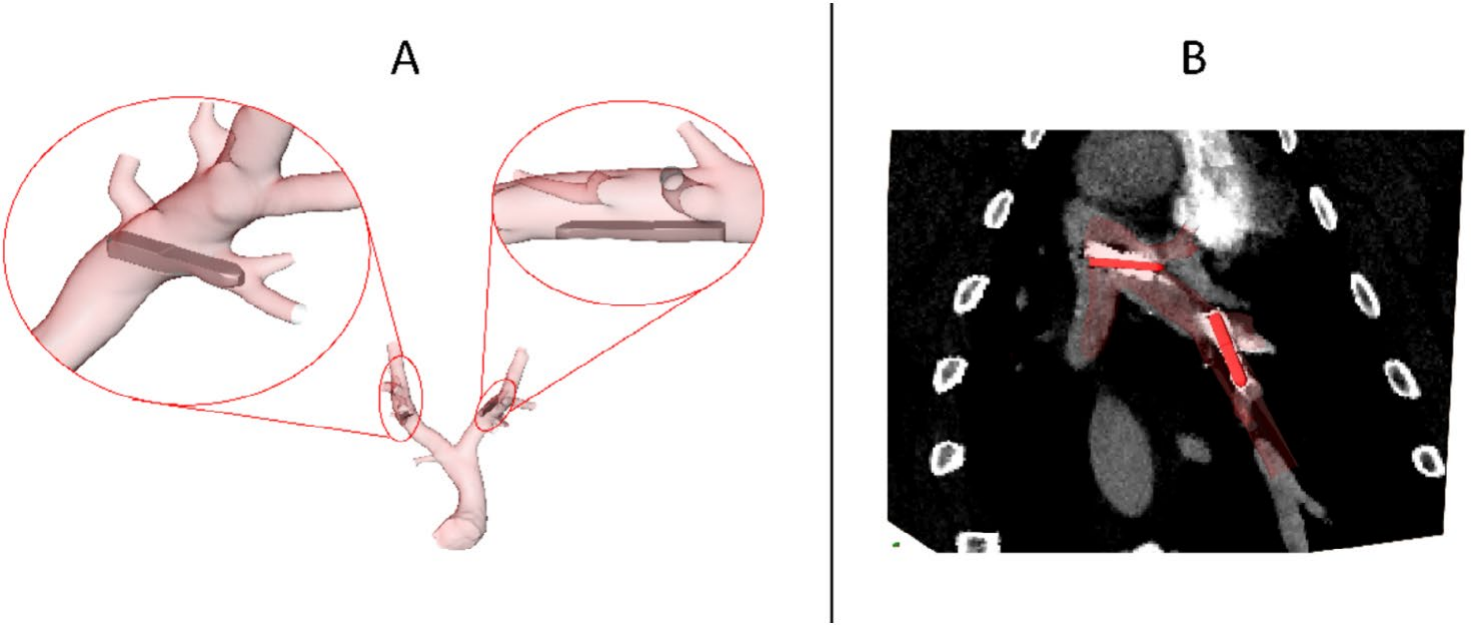
(A) Example of a porcine case (9), which features one optimally (top right) and one non‐optimally implanted sensor (top left). Non‐optimally implanted sensors are characterized by either protruding into a side branch or lying perpendicular to the vessel centerline. (B) CT image data showing the in vivo sensor position used for probe positioning reference.

Finally, 20 PAPS were virtually implanted into the human PA, aiming to mimic pairwise implantation sites into the porcine PA, including optimally and non‐optimally implanted devices. Here, an optimal device position was defined as the entirety of the device lying adjacent to the vessel wall. In contrast, non‐optimal device positions were indicated by skewed devices lying across the cross‐section of the vessel, as well as devices being positioned entirely or partly in a side branch of the PA. Figure 1 shows all 10 human and 10 porcine PA with one PAPS device implanted into the LPA and one into the RPA, while Figure 2 shows an example of an optimally and a non‐optimally implanted sensor. In total, 8 of the 20 sensors were found to be implanted in a non‐optimal position in the porcine cohort. Cases that had non‐optimally implanted sensors are marked in Figure 1.

### 2.1 Computational Fluid Dynamics Analysis

**FIGURE 3:**
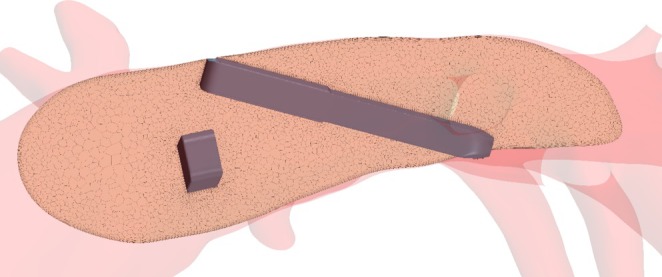
Mesh structure, exemplary depicted for porcine case 1 in the LPA region.

Transient blood flow simulations were performed using STAR‐CCM+ (15.04, Siemens PLM, Plano, Texas). The software was used to create a computational mesh as well as to solve the governing equations of flow using a finite‐volume based solver. The fluid domain was discretized using polyhedral cells with a base size of 0.75 mm, which resulted in approximately 1 million cells (2.5 million vertices) per case, depending on vessel size. The mesh and time step size were set based on a mesh convergence study which yielded the above size as the best balance between accuracy and computational demand. To accurately resolve near‐wall flow, a mesh boundary layer with six prism layers (initial height 0.02 mm, growth rate 1.3, total height of 0.3 mm) was created at the vessel and sensor walls. Figure 3 shows a cross‐section through the LPA of porcine case one to illustrate the mesh structure.

**FIGURE 4:**
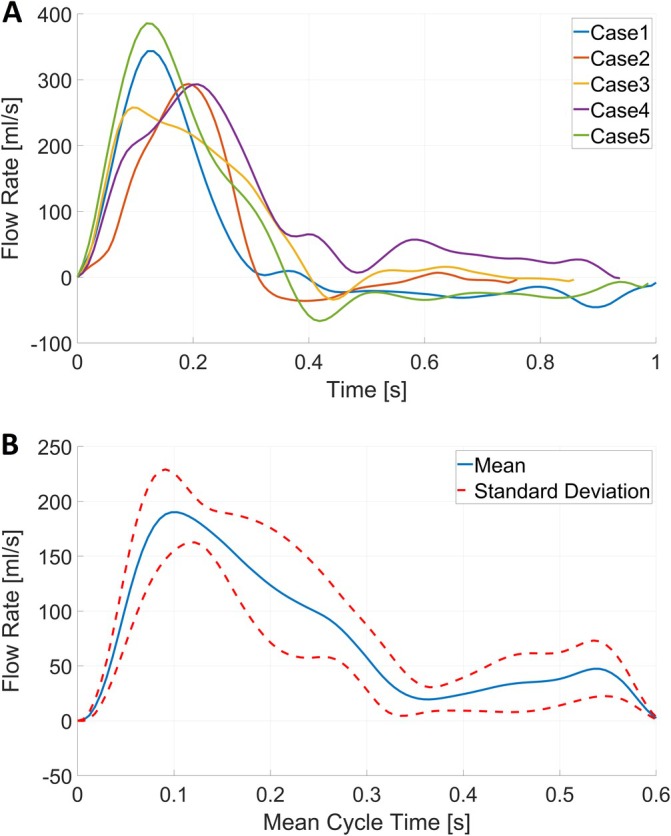
(A) Five MPA flow rate curves generated to simulate flow in human PA. (B) Average with standard deviations flow rate waveform representing 10 synthetically generated curves to simulate flow in porcine PA.

Blood was modeled as an incompressible fluid with a density of 1050 kg/m^3^ and a shear‐rate dependent viscosity following a Carreau‐Yasuda model with coefficients described here [26]. Given the high Reynolds numbers (> 2000) expected at peak systolic flow rates, a *k*‐*ω* SST turbulence model is used to account for turbulent effects. The vessel wall was assumed to be rigid, and a no‐slip boundary condition was applied. A constant time step of 1 ms was used for all simulations. The inlet boundary condition at the main PA (MPA) inlet surface prescribed a mass flow rate as a function of physical time at the MPA boundary using a plug profile. These flow rate curves were obtained from an MPA flow rate model, which is based on a large set of in vivo measured, time‐resolved pulmonary artery flows of pigs [23] and humans (unpublished data). Individual hemodynamics are accounted for by using subject‐specific heart rates (HR) and cardiac outputs (CO) to produce each flow rate curve. Subject‐specific HR and CO values were obtained from clinical measurements for the human cases. HR and CO for the porcine cases were not available but were obtained from an empirical model, which relates body weight (which was known for each case) to HR and CO [23]. Figure 4A shows the generated flow rate waveforms for all five human cases, whereas Figure 4B shows an averaged flow rate waveform used in simulations of the porcine PA. At all outlets, a constant pressure boundary condition of 20 mmHg was used. Furthermore, a constant low turbulence intensity of 5% was assumed at the inlet.

CFD results were post‐processed using MATLAB R2021a (MathWorks, USA). Three parameters were evaluated: time‐averaged wall shear stress (TAWSS) and OSI, which are parameters associated with a risk of thrombus formation [9, 27, 28] as well as pressure drop across the PAPS. To evaluate WSS and OSI, we calculated surface‐averaged values as well as areas with low WSS (< 0.4 Pa) and high OSI (> 0.3). The pressure drop caused by the device was calculated as a difference between cross‐section averaged static pressure measured 10 mm downstream and 10 mm upstream of the device. This parameter can be seen as a measure of the impact the device has on the intra‐arterial hemodynamics caused by the device and is not directly associated with the thrombus risk. Normally distributed parameters are described as mean ± standard deviation; otherwise, median with (interquartile range) is used. Normality of distribution was assessed using a Shapiro–Wilk test. Statistical analysis was performed using IBM SPSS Statistics 28 (IBM, USA).

## 3 Results

### 3.1 Comparison Between Human and Porcine PA

Comparing morphometric parameters between porcine and human PA used in both in silico studies (see also Table 1) revealed significantly larger diameters in human RPA (20.5 ± 1.6 mm vs. 14.8 ± 2.0 mm, *p* < 0.001; Student's *t* test) and LPA (17.9 ± 1.3 mm vs. 13.6 ± 1.7, *p* = 0.003 Mann–Whitney test) segments, whereas no significant differences were found for the MPA diameters (23.9 ± 2.2 mm vs. 27.4 ± 4.4 mm, *p* = 0.053 Student's *t* test) and bifurcation angles (86° ± 9° vs. 80° ± 7°, *p* = 0.221; Student's *t* test).

Comparing hemodynamic boundary conditions for porcine and human PA simulations, significantly lower HR in humans with 66.8 ± 7.9 bpm vs. 99.7 ± 3.0 bpm in pigs (*p* < 0.001, Student's *t* test) were found, whereas no significant differences in CO were found (4.0 ± 1.3 L/min vs. 4.7 ± 0.2 L/min, *p* = 0.137, Student's *t* test). Furthermore, the peak systolic flow rate was significantly higher in the human MPA (332.0 ± 52.0 mL/s vs. 216.0 ± 27.3 mL/s, *p* < 0.001, Student's *t* test, see also Figure 4).

### 3.2 Comparison of Hemodynamics Before and After PAPS Implantation

**FIGURE 5:**
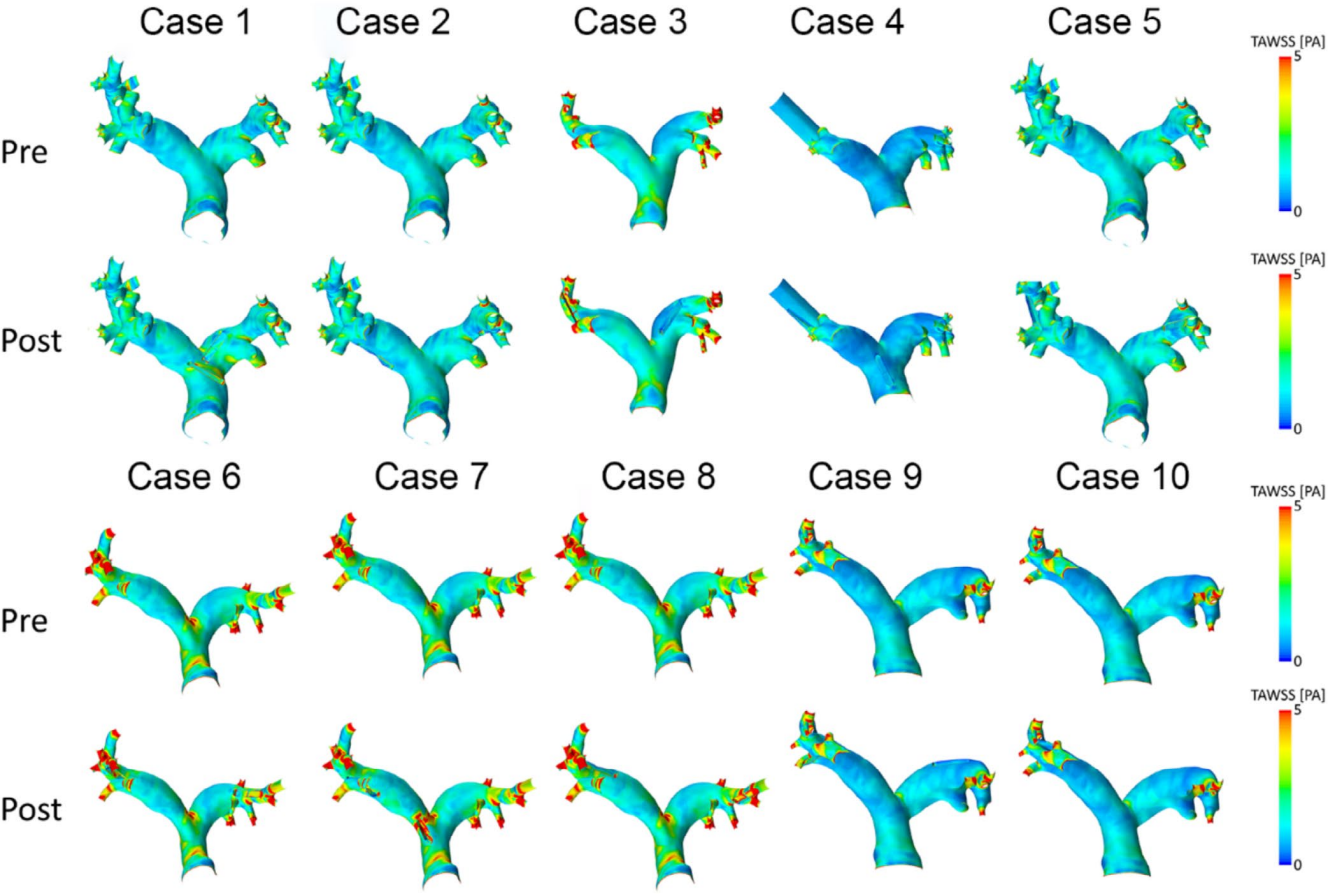
Spatial distributions of time‐averaged wall shear stress (TAWSS) values before (pre) and after (post) virtual implantation of two pulmonary artery pressure sensors (PAPS) in each human PA.

**FIGURE 6:**
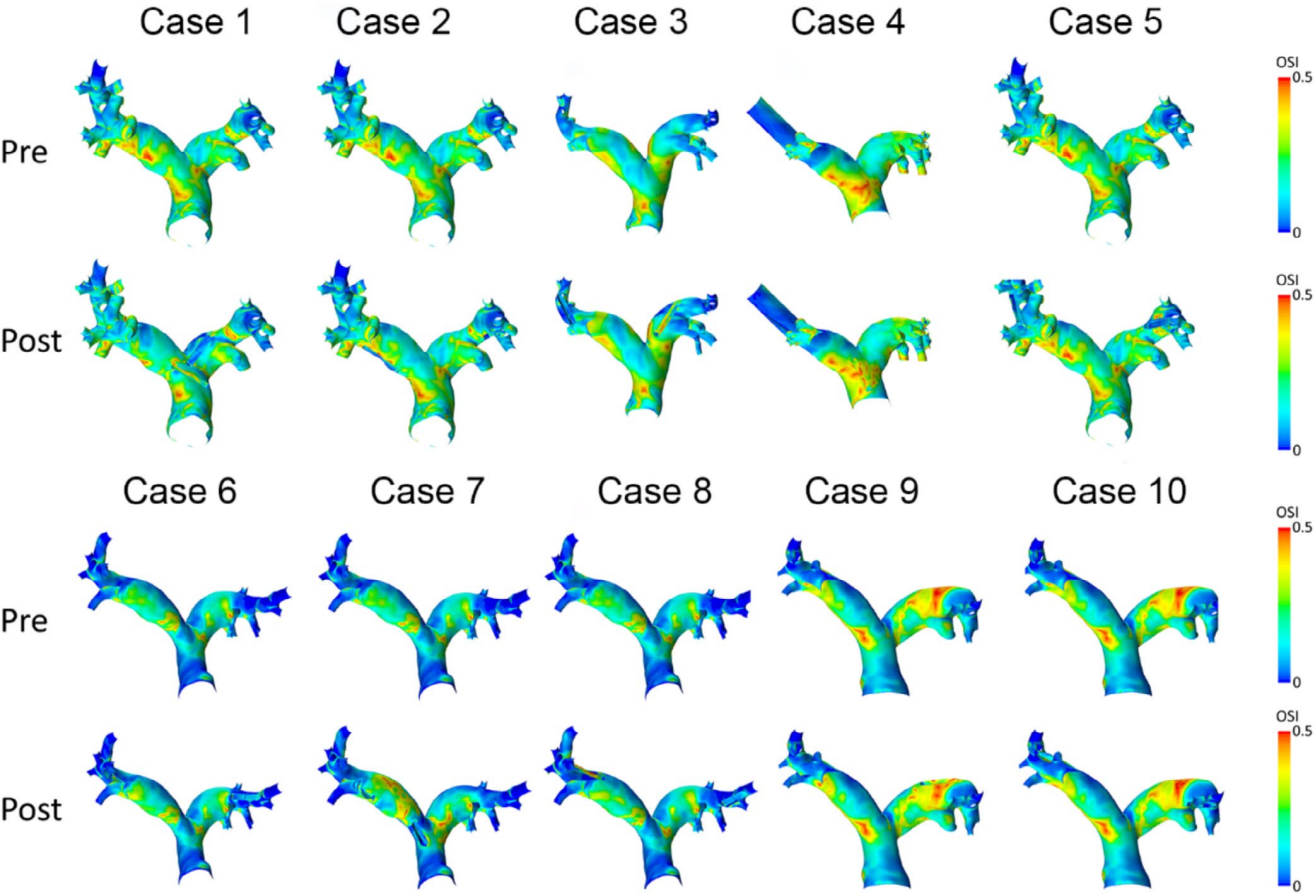
Spatial distributions of oscillating shear index (OSI) values before (pre) and after (post) virtual implantation of two pulmonary artery pressure sensors (PAPS) in each human PA.

Figures 5 and 6 show distributions of TAWSS and OSI in the human and porcine PA before and after sensor implantation. After device implantation, only cases 6 and 8 showed a potentially relevant increase of TAWSS by 0.18 Pa (7%) and 0.16 Pa (11%), respectively. Overall, TAWSS was found to be significantly higher after PAPS implantation (pre: 1.38 [1.11] Pa; post: 1.44 [1.15] Pa, *p* = 0.009, Wilcoxon test), the difference of 0.06 Pa is clinically neglectable (see Figure 5). Changes in OSI remained below 0.01 for all cases, and no significant difference was found (pre: 0.16 [0.07]; post: 0.15 [0.07], *p* = 0.541, Wilcoxon test). In the porcine PA, both TAWSS and OSI were slightly higher after the implantation but remained below any clinically relevant changes (< 0.05 Pa for TAWSS, 0.005 for OSI).

In the human PA, TAWSS was lower compared to the porcine PA both pre‐ and post‐implantation (pre: 1.38 [1.11] Pa vs. 2.35 ± 0.47 Pa, *p* = 0.028; post: 1.44 [1.15] Pa vs. 2.45 ± 0.49 Pa, *p* = 0.028, Mann–Whitney‐*U* test), whereas OSI was significantly higher in the human PA (pre: 0.16 [0.07] vs. 0.08 ± 0.17, *p* < 0.001; post: 0.15 [0.07] vs. 0.08 ± 0.16, *p* < 0.001, Mann–Whitney‐*U* test). This difference is explained by significantly larger diameters of the human PA, whereas no significant differences were found for CO.

Furthermore, the device implanted into the human PA caused a relatively low average pressure drop of 0.8 ± 0.8 mmHg, which does not (*p* = 0.602, Student *t* test) differ significantly from the pressure drop calculated in porcine PA, with 0.7 ± 1.1 mmHg.

In summary, differences in hemodynamics between porcine and human PA have been found, indicated by lower TAWSS and higher OSI in human PAs. Despite these differences, device implantation resulted in no relevant shifts of hemodynamic parameters towards higher risks for thrombosis in either the porcine or in the human PA.

### 3.3 Comparison of Hemodynamics Between Optimal vs. Non‐Optimal PAPS Implantations

Comparison of hemodynamics between optimally vs. non‐optimally implanted devices within the human PA revealed no significant difference for TAWSS on the PAPS surface (1.7 ± 0.9 Pa vs. 2.8 ± 1.7 Pa, *p* = 0.081, Student's *t* test) and no significant differences for OSI on the PAPS surface (0.19 ± 0.09 vs. 0.12 ± 0.04, *p* = 0.056, Student's *t* test).

Consequently, significantly smaller areas affected by low WSS values on the PAPS surface (< 0.4 Pa) were found in non‐optimal sensors (optimal vs. non‐optimal: 0.28 [0.25] cm^2^ vs. 0.10 [0.12] cm^2^, *p* = 0.011, Mann–Whitney test) and no significant differences (0.62 [1.00] cm^2^ vs. 0.36 [0.72] cm^2^, *p* = 0.247, Mann–Whitney test) for areas with high OSI (> 0.3).

Similar results were found in the study focusing on the porcine PA published earlier [4]: TAWSS—2.55 ± 0.56 Pa vs. 3.85 ± 0.71 Pa (*p* < 0.001) and OSI—0.12 ± 0.08 vs. 0.09 ± 0.03 (*p* = 0.263). Despite the significantly lower TAWSS and significantly higher OSI found for PAPS in human PA compared to porcine PA, both CFD results (porcine and human) found no higher risk of thrombus formation for non‐optimally implanted PAPS.

## 4 Discussion

In silico modelling can assess parameters that cannot be measured directly during in vivo pre‐clinical studies, for example due to poor imaging quality, invasiveness of measurement methods, or the lack of measurement methods. Thus, assessing device effects, such as changes in hemodynamics after device implantation, is challenging. However, the knowledge of the interaction between implantable devices and the cardiovascular system is crucial for the assessment of safety and efficacy, and thus for a successful device development and approval process.

Accordingly, in silico studies investigating the change of hemodynamics associated with an implantation or treatment procedure of the pulmonary artery are known for different devices including a reducer stent to be percutaneously implanted in an enlarged PA [29], a pulmonary autograft to be used for the treatment of aortic valve disease in young patients [30], stenting of the pulmonary artery stenosis in congenital heart defects [31] or stenting of the PA branch [32].

So far, no in silico studies investigating the hemodynamic effect of PAPS devices on PA hemodynamics have been published. A potential reason for this is that PAPS devices are rather new. However, for other novel implantable pressure sensor designs, in silico studies exist. Manavi et al. investigated the impact of a novel anchoring system of a pressure sensor on hemodynamics [9]. However, they proposed an alternative solution by implantation of a pressure sensor into the Vena Cava instead of the pulmonary artery.

As we noted in the introduction, the study presented here was motivated by a controversial consideration regarding the suitability of pre‐clinical animal testing for the evaluation of the safety and efficiency of novel medical devices. These doubts [3, 4, 5] are well‐founded considering differences between species regarding, in our case, major parameters affecting PA hemodynamics: geometric factors including shape (e.g., number of side branches) and major size (diameters, lengths and bifurcation angle) parameters as well as hemodynamic factors such as flow and heart rates [25].

To solve this problem, we proposed an in silico approach to simultaneously assess differences between animal and human hemodynamic conditions as well as investigating the transferability of the animal study findings to human application. The use of CFD in animal models to assess hemodynamic metrics such as WSS or blood velocity profiles could provide better insights into the pathology, assist treatment procedures, predict disease progression as well as improve the generalizability of findings [33]. Using animal models is advantageous because they provide better controlled conditions [33]. On the other hand, the use of animal models is associated with some obstacles, for example, the difference of animal vascular anatomy from human anatomy [33, 34]. Respectively, the translation of findings for animal CFD studies towards human conditions must be done with care.

The focus of our CFD study approach was to assess the potential increase in thrombus risk resulting from hemodynamic changes caused by an implanted sensor. The in silico enhanced chronic animal study found no such increased risks, not even after non‐optimal device implantation. Neither have relevant alterations in the calculated parameters associated with thrombosis been observed, nor have there been any signs of thrombosis or lung embolism observed at the end of the chronic animal study. Furthermore, these observations agree well with the overall very low adverse event rate reported for existing PAPS devices, such as very low incidence rates of embolic events ranging from 0% to 1% reported from the relevant PAPS trials [35].

The results of this study preliminarily indicate evidence for the safety of the PAPS device in a human application regarding the thrombosis risk. First, we found that the overall hemodynamics between pigs and humans are comparable, indicating that the animal model, while not an ideal match regarding anatomy and flow conditions, is at least close with respect to the relevant parameter ranges. Second, no relevant hemodynamic changes due to sensor implantation were found in either the animal or in the human PA. Thus, the in silico model was used to evaluate the transferability of the animal study results towards human conditions. It must be noted that these findings can only be considered as a necessary condition for evaluating device safety, but not a sufficient one and do not imply that further studies such as in vitro thrombogenicity tests can be omitted. Of course, not all in vivo risk factors associated with an implantable device can be assessed through in silico studies. On the other side, not all risk factors can be measured in vivo—for example, WSS and OSI parameters. Thus, a combination of in vivo, ex vivo, in vitro, and in silico studies, as currently used by many medical device companies, seems to be an optimal approach for device development, approval, and clinical translation aiming to reduce risks associated with device safety and efficacy.

Unfortunately, there is a lack of studies combining animal CFD with human CFD similar to our study. Our PubMed research found only one CFD study, which compared morphometric and hemodynamic parameters between swine and human coronary artery focusing on atherosclerosis [36]. Note that hemodynamic parameters of interest for thrombosis and atherosclerosis research, including such parameters as WSS and OSI, are the same. In contrast to our study, a comparison for coronary arteries found more similarities for both morphometric and geometric parameters compared to our study. Among four investigated geometric parameters, only shape index describing cross‐section eccentricity was significantly different between swine and human models. Similar to our study, a significantly higher peak flow rate was found in the left anterior descending artery (LAD) of swine. In swine LAD, also a significantly higher OSI was found, whereas no significant differences were found for time‐averaged WSS, helicity, or relative residence time. In general, this as well as our study underlines the importance of CFD studies comparing morphometry and hemodynamics among different species.

## 5 Limitations

This in silico study is associated with some limitations of the numerical model. Simulated flow rate waveforms at MPA inlets were generated synthetically since no patient‐specific data were available and no possible changes in flow rates after PAPS implantation were considered. This is motivated by the fact that PAPS causing a pressure drop of less than 1 mmHg can be considered a neglectable PA flow obstacle. Flow simulations were also done assuming rigid walls. We consider this assumption non‐critical since HF patients are associated with stiffer PA [37] and because a study of Kong et al. found only minor differences between TAWSS distributions in the PA simulated with a rigid wall and using a fluid–structure interaction approach [38]. A validation study of the proposed CFD model using an experimental study or in vivo 4D flow magnetic resonance imaging (MRI) is desirable. A validation was not done in the frames of our study since earlier published studies, which used CFD models of the same complexity as ours, approved the CFD accuracy for the pulmonary artery hemodynamics analysis [32, 39].

The analysis of PA hemodynamics was limited to OSI and TAWSS, even though a set of other parameters was proposed in the literature as potential thrombus formation risk parameters. We decided on OSI and TAWSS since these are the most frequently used [40, 41, 42, 43]. It is also necessary to note that the assessment of hemodynamic parameters in a CFD study is affected by model inputs, which are inherently uncertain [44]. Thus, a full analysis of the sensitivity and uncertainty of the CFD model is desirable but is computationally expensive and usually requires a separate study [44, 45].

Finally, some limitations are associated with the study design, including the modelling of 20 PAPS in 10 human pulmonary arteries, selection of implantation sites, and the use of only five human PA for the human in silico study. All these limitations are consequences of our study objective, aiming to investigate the transferability of the animal in silico PAPS study findings to human conditions. Thus, the human PAPS study aimed to mimic the PAPS study performed in pigs. Two PAPS were implanted in each porcine PA, aiming to reduce the number of animals in the study. The intended least possible difference between porcine and human geometric parameters of the PA, as well as the limited number of available human PA geometries with low variability of PA shapes of 10 animals, results in the use of only five human PA geometries for 10 porcine PA geometries, thus reducing the variability of pre‐implantation conditions. The decision to achieve the least possible difference between human and porcine cohorts is explainable since the aim of each pre‐clinical animal study is to replicate human conditions as closely as possible. A comparative analysis of morphometric parameters describing PA geometry showed overlapping ranges for most investigated parameters and consequently the potential ability to replicate human conditions [25]. However, despite the intended similarity between porcine and human cohorts, we found that a combination of morphometric and hemodynamic conditions differs between both cohorts. Since both in silico studies result in the same findings, we consider the transferability between both studies as feasible but limited by a designed similarity between porcine and human PA. The power of the transferability finding of our study can be, however, increased in future in silico studies by a selection of a human cohort, which would, oppositely to our study, be less similar to the animal study cohort.

As a result of the study design aiming to mimic the PAPS implantation configurations of an animal study including optimally and non‐optimally implanted sensors, PAPS were virtually implanted in human PA segments of significantly higher diameters than sensors implanted in porcine PA (18.47 ± 5.70 mm vs. 12.18 [3.49] mm, *p* < 0.001, Wilcoxon test). Note that the target implantation site of the PAPS is about 10.0 mm. Only four sensors were implanted in human PA according to this requirement, whereas in porcine PA altogether 14 sensors fulfilled this requirement.

We consider all these limitations as non‐critical regarding our findings since all simulated configurations represent possible real situations and at the same time reflect anatomical and hemodynamic differences between both species. However, the results of this in silico study should be considered with caution and a future in silico study with a significantly higher number of simulated PA shapes is necessary.

## 6 Conclusion

In this human in silico study, we were able to mimic the animal in silico study, which in turn enhanced the chronic animal study by modeling hemodynamic parameters, which cannot be measured in vivo. Despite inter‐species differences, both in silico studies with porcine and human PA found no relevant changes in hemodynamics after pressure sensor implantation. This is also valid for PAPS, which were non‐optimally implanted. Based on a comparison of results between animal and human in silico studies, the potential ability and advantage of the in silico approach to translate results of preclinical research to human/clinical investigations were shown.

## Author Contributions

J.B. and L.G. segmented and reconstructed surface geometries. A.S. performed virtual implantations of PAPS and prepared final geometries for CFD simulations. J.R. elaborated criteria for PAPS implantation. P.Y. performed CFD analysis and post‐processing of CFD data. L.G. performed statistical analysis. L.G., A.A., and T.K. elaborated the study concept. L.G., P.Y., and J.B. prepared the original draft of the manuscript. A.A. and J.R. reviewed and edited the manuscript. All authors contributed to the article and approved the submitted version.

## Ethics Statement

The authors have nothing to report.

## Conflicts of Interest

J.R. is an employee and A.A. is a former employee of Biotronik. The other authors declare no conflicts of interest.

## Data Availability

The data that support the findings of this study are available on request from the corresponding author. The data are not publicly available due to privacy or ethical restrictions.
